# Highly Sensitive Non-Dispersive Infrared Gas Sensor with Innovative Application for Monitoring Carbon Dioxide Emissions from Lithium-Ion Battery Thermal Runaway

**DOI:** 10.3390/mi16010036

**Published:** 2024-12-29

**Authors:** Liang Luo, Jianwei Chen, Aisn Gioronara Hui, Rongzhen Liu, Yao Zhou, Haitong Liang, Ziyuan Wang, Haosu Luo, Fei Fang

**Affiliations:** 1 Department of Engineering Mechanics, Tsinghua University, Beijing 100084, China; medinathu@163.com; 2 Shanghai Institute of Ceramics, Chinese Academy of Sciences, Shanghai 201800, China; hsluo@mail.sic.ac.cn; 3 Einsteck Inc., Palo Alto, CA 94305, USA; einsteck@126.com; 4 School of Instrument and Electronics, North University of China, Taiyuan 030051, China; 13027020313@163.com; 5 School of Modern Post, Xi’an University of Posts and Telecommunications, Xi’an 710061, China; vejango123@163.com; 6 College of Materials Science and Technology, Beijing Forestry University, Beijing 100083, China; lianghaitong@bjfu.edu.cn; 7 School of Integrated Circuits, Southeast University, Nanjing 214135, China; 230228414@seu.edu.cn

**Keywords:** non-dispersive infrared, pyroelectric, carbon dioxide gas sensor, lead niobium magnesium titanate

## Abstract

The safety of power batteries in the automotive industry is of paramount importance and cannot be emphasized enough. As lithium-ion battery technology continues to evolve, the energy density of these batteries increases, thereby amplifying the potential risks linked to battery failures. This study explores pivotal safety challenges within the electric vehicle sector, with a particular focus on thermal runaway and gas emissions originating from lithium-ion batteries. We offer a non-dispersive infrared (NDIR) gas sensor designed to efficiently monitor battery emissions. Notably, carbon dioxide (CO_2_) gas sensors are emphasized for their ability to enhance early-warning systems, facilitating the timely detection of potential issues and, in turn, improving the overall safety standards of electric vehicles. In this study, we introduce a novel CO_2_ gas sensor based on the advanced pyroelectric single-crystal lead niobium magnesium titanate (PMNT), which exhibits exceptionally high pyroelectric properties compared to commercially available materials, such as lithium tantalate single crystals and lead zirconate titanate ceramics. The specific detection rate of PMNT single-crystal pyroelectric infrared detectors is more than four times higher than lithium tantalate single-crystal infrared detectors. The PMNT single-crystal NDIR gas detector is used to monitor thermal runaway in lithium-ion batteries, enabling the rapid and highly accurate detection of gases released by the battery. This research offers an in-depth exploration of real-time monitoring for power battery safety, utilizing the cutting-edge pyroelectric single-crystal gas sensor. Beyond providing valuable insights, the study also presents practical recommendations for mitigating the risks of thermal runaway in lithium-ion batteries, with a particular emphasis on the development of effective warning systems.

## 1. Introduction

The rapid growth of new energy vehicles has driven a surge in demand for high-energy-density batteries. However, this increased demand also heightens the risk of battery malfunctions [1,2]. When lithium-ion batteries fail, they can generate twice as much heat per mile as gasoline engines. The severe consequences of thermal runaway, such as fires and explosions, pose critical safety hazards, underscoring the necessity for effective thermal runaway detection [3,4,5]. Thermal runaway in lithium-ion batteries is a major safety concern in the electric vehicle industry. The United Nations’ Global Electric Vehicle Safety Technical Regulation mandates that a warning be issued at least five minutes before a dangerous situation is caused by thermal runaway, highlighting the pressing need for an effective thermal runaway detection system [6,7].

Existing battery thermal runaway detection methods typically rely on voltage, current, or surface temperature measurements. Similarly, methods that monitor changes in battery internal resistance also rely on voltage measurement and face signal suppression issues in parallel circuits, so using only voltage for thermal runaway detection has become challenging. Therefore, a highly reliable and responsive detection method is needed. Various gas emissions, including carbon dioxide (CO_2_), carbon monoxide (CO), and volatile organic compounds (VOCs), are often precursors to thermal runaway in lithium-ion batteries [8]. During thermal runaway, new energy vehicle batteries release significant amounts of gas. After the event, LiNi_x_Co_y_Mn_z_O_2_ (NCM) batteries produce more gas than LiFePO_4_ (LFP) batteries [9]. When a battery malfunctions and subsequently catches fire or explodes, the released gases can lead to catastrophic consequences in electric vehicles and electrochemical energy storage systems. Therefore, gas sensors are essential for the rapid and accurate detection of these harmful emissions.

The primary gas components released during the thermal runaway of NCM and LFP batteries include CO_2_, CO, hydrogen (H_2_), methane (CH_4_), and ammonia (NH_3_) [9]. These gases can serve as key indicators for detecting thermal runaway in batteries, offering essential data to support the future development of lithium-ion battery technology. Among these gases, CO_2_ is the dominant component, making up 62.7–96.6% of the volume [10]. The release of CO_2_ is the first sign of thermal runaway in lithium-ion batteries. The early detection of CO_2_ allows for prompt action, preventing major failures and enhancing battery safety. Real-time monitoring of CO_2_ emissions continuously assesses battery health, enabling immediate detection and swift corrective measures to reduce the risk of dangerous incidents. Therefore, the rapid and accurate detection of CO_2_ gas is of paramount importance. Previous studies on detecting thermal runaway in battery storage facilities have demonstrated that gas detection methods targeting CO_2_ concentrations respond more quickly than monitoring the temperature of the storage tank surface [11]. This underscores the critical role of gas sensors in early-warning systems, as they offer faster and more reliable indications of thermal runaway events compared to traditional temperature-based approaches. By analyzing CO_2_ concentration curves, the battery management system can monitor real-time gas concentration changes around or within the battery to detect abnormalities. As a result, high-sensitivity CO_2_ sensors are commonly employed in critical areas of battery management and safety assurance for continuous monitoring [8,12].

Due to its early presence during the initial exhaust, CO_2_ is considered an excellent target gas for detection. In new energy vehicle battery packs, gas sensors include electrochemical sensors, semiconductor sensors, chemical sensors, and non-dispersive infrared (NDIR) sensors [13,14,15,16,17]. Electrochemical gas sensors are sensitive to multiple gases, can experience up to a 15% drift annually, and may be damaged in low-humidity environments. Semiconductor gas sensors are prone to contamination and signal drift and have high power consumption. Chemical gas sensors tend to drift and degrade rapidly, requiring frequent replacements and thereby increasing usage costs. However, NDIR CO_2_ sensors are favored due to their good selectivity, reasonable cost, minimal sensor drift, and long lifespan, making them a feasible choice for gas detection [18,19]. Many gases absorb specific wavelengths of infrared light, and the gas concentration can be calculated by measuring how much light is absorbed at these wavelengths by using the Lambert–Beer Law. The commonly used wavelength for CO_2_ detection is 4.26 μm, which is not absorbed by other common gases or water vapor. This wavelength specificity gives NDIR sensors high selectivity for CO_2_ gas and largely avoids any cross-sensitivity issues with other gases [20].

This paper presents an NDIR gas sensor designed for monitoring CO_2_ concentrations and early warning of lithium-ion battery failure. The sensor system includes a high-performance pyroelectric CO_2_ infrared detector, a MEMS infrared light source, a gas chamber optical path, a data acquisition and A/D conversion module, and a display module. By analyzing the failure process of lithium-ion batteries, a CO_2_ gas sensor suitable for tracking these events was developed, with a test chamber used to simulate the battery failure process and enable real-time CO_2_ concentration monitoring. The results show that using a high-performance pyroelectric infrared gas detector with a lead niobium magnesium titanate [(1−*x*)Pb(Mg_1/3_Nb_2/3_)O_3_-*x*PbTiO_3_, PMNT] single crystal as the sensitive element significantly enhances the detection performance over commercial lithium tantalate (LiTaO_3_, LT). Highly sensitive PMNT single-crystal CO_2_ sensors accurately identify thermal runaway in new energy batteries by detecting even minor changes in CO_2_ levels. This improvement notably increases CO_2_ detection accuracy, lowers the minimum detection threshold, and reduces the response time, providing robust support for lithium battery failure monitoring and early warning [20,21].

## 2. Principle of NDIR Gas Sensor Using Pyroelectric Infrared Detector

**Figure 1:**
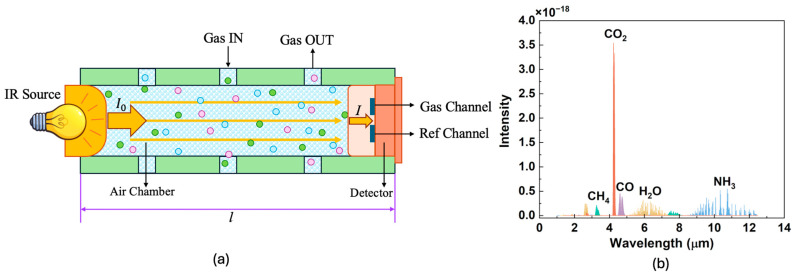
(**a**) Schematic diagram of the NDIR gas sensor and Lambert–Beer law; (**b**) infrared absorption peaks of different kinds of gases.

Figure 1a shows the detection model and physical image of the NDIR method gas sensor. On the left side, the infrared light source emits radiation across all wavelengths, and the chamber directs the emitted light toward the detector on the right side. The detector consists of two channels, each with a narrowband filter above the thermoelectric-sensitive element. One channel is the gas-sensing channel, which is designed to be absorbed only by the measured gas, while the other channel serves as a reference channel, unaffected by common gases. By measuring the reduction in radiation within the gas-sensing channel, the Lambert–Beer law can be applied to determine the gas concentration. When a parallel infrared light with an initial intensity of *I* passes through the gas medium, the outgoing light intensity is reduced to $I_{0}$ due to selective absorption. In this context, α(λ) represents the absorption coefficient at wavelength λ, c denotes the concentration of the gas being measured, and *l* is the optical path length of the gas chamber. The relationship is mathematically expressed as follows [22,23]:

(1) $$I(\lambda )=I_{0}(\lambda )\times e^{-\alpha (\lambda )cl}$$

By comparing the intensity of light reaching the detector through the gas-sensing channel to the intensity in the reference channel, the concentration of gas can be accurately calculated, as the reduction in radiation is proportional to the amount of gas present in the chamber.

(2) $$\frac{dI}{I}=-kcdx$$

(3) $$\int_{I_{0}}^{I}\frac{dI}{I}=-kc\int_{0}^{l}dx$$

(4) $$I=I_{0}e^{-kcl}$$

Figure 1b shows the infrared spectra of various gases. CO_2_ has an absorption peak in the 4.26 μm, where the absorption coefficient is highest and the half-width is relatively narrow, and there is no interference from other gases. Additionally, the mid-infrared range from 3–5 μm offers a wide selection of infrared sources and narrow-band filters. Consequently, the 4.26 μm absorption peak is commonly used for monitoring the CO_2_ gas concentration [24].

**Figure 2:**
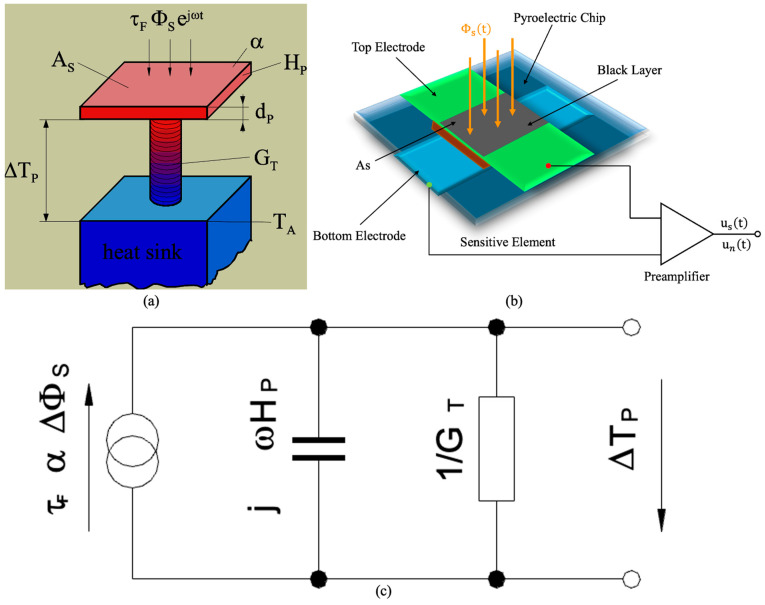
(**a**) Thermal model of pyroelectric infrared detectors. (**b**) Structure and circuit of pyroelectric infrared detectors. (**c**) Equivalent circuit of pyroelectric infrared detectors.

In Figure 2a, the radiation flux $\Phi_{S}$ striking the detector window first passes through an optical filter with transmission ($\tau_{F}$) and then reaches the pyroelectric chip. This causes a temperature increase in the pyroelectric chip ($\Delta T_{P}$), which is proportional to its optical absorption ($A_{s}$) and inversely proportional to its heat capacity ($H_{P}$). Meanwhile, the applied heat dissipates into the environment, primarily through the chip holder, at temperature ($T_{A}$), characterized by thermal conductance ($G_{T}$). These two competing processes—heating of the thermal mass and cooling via heat conduction—are governed by thermal conductivity, a key parameter in the heat conduction equation. This equation is a partial differential equation that typically needs to be solved while considering boundary and initial conditions [25].

When the pyroelectric chip-sensitive element (with an absorption rate of *α*) is exposed to sinusoidal modulated infrared radiation,

(5) $$\Phi (t)=\Phi_{0}e^{i\omega t}$$

The temperature of the sensitive element changes ΔT due to the increase in internal energy. However, a portion of this energy is lost to the environment through thermal conduction (with thermal conductance $G_{T}$). Therefore, the following thermal equilibrium equation can be established:

(6) $$H_{p}\frac{d(\Delta T)}{dt}+G_{T}(\Delta T)=\alpha \Phi_{0}e^{i\omega t}$$

where $H_{p}$ represents the heat capacity of the PMNT-sensitive element.

(7) $$H_{p}=C_{V}\cdot A_{s}\cdot d$$

$C_{V}$ is the specific heat per unit volume of the PMNT-sensitive element.

Since the differential form of the basic circuit equation is similar, a thermoelectric analogy can be made to convert the thermal model into the equivalent circuit shown in Figure 2c. Using this equivalent circuit, the temperature variation ΔT of the sensitive element under sinusoidally modulated infrared radiation can be obtained:

(8) $$\Delta T(t)=\frac{\alpha \Phi_{0}}{G_{T}+i\omega H_{p}}e^{i\omega t}=\frac{\alpha \Phi (t)}{G_{T}\left(1+i\omega \tau_{T}\right)}=\frac{\alpha \Phi (t)}{G_{T}\sqrt{1+{\left(\omega \tau_{T}\right)}^{2}}}$$

where $\tau_{T}$ represents the thermal time constant,

(9) $$\tau_{T}=H_{p}/G_{T}$$

The thermal time constant is a fundamental parameter in the study of thermal systems, representing the characteristic time it takes for a system to respond to a change in temperature. It quantifies how quickly a system can adjust to thermal disturbances and reach a new thermal equilibrium. The thermal time constant in NDIR sensors is influenced by factors such as thermal capacitance, thermal conductance, material properties, sensor design, heat transfer methods, temperature stability, and thermal inertia. These elements determine how the sensor responds to temperature changes, maintains stability, and ensures accurate gas detection. By understanding and optimizing the thermal time constant, we can design NDIR sensors that achieve the right balance of response speed, stability, sensitivity, and energy efficiency for specific applications.

In Figure 2b, the pyroelectric infrared detector consists of a pyroelectric material that generates a current when it absorbs infrared radiation. The amount of current generated is proportional to the rate of temperature change in the material, which is influenced by the intensity of the absorbed infrared radiation. This current is then used to detect and measure the presence and concentration of gases based on their infrared absorption properties [25].

The generated current $i_{p}$ can be expressed as:

(10) $$i_{p}=A_{s}\frac{dP_{s}}{dt}=A_{s}p\frac{d(\Delta T)}{dt}=\frac{A_{s}p\omega \alpha }{G_{T}}\frac{\Phi (t)}{\sqrt{1+{\left(\omega \tau_{T}\right)}^{2}}}$$

The performance of pyroelectric infrared detectors is typically measured by parameters such as noise ($u_{n}$), responsivity ($R_{V}$), noise equivalent power (*NEP*), and specific detectivity ($D^{*}$).

(11) $$R_{V}=u_{s}/\Phi$$

(12) $$NEP=\frac{u_{n}}{R_{V}}$$

(13) $$D^{*}=\frac{\sqrt{A_{s}B}R_{V}}{u_{n}}$$

In these equations, $u_{s}$ is the response voltage, Φ represents the radiation power, $u_{n}$ is the root mean square of the noise power spectral density S(ω), $A_{s}$ is the area of the sensitive element, and B is the amplifier bandwidth.

*NEP* reflects the minimum infrared radiation power that the detector can detect, while $D^{*}$ expresses the signal-to-noise ratio in relation to the detector’s unit area, amplifier bandwidth, and radiation power. A detector with a higher $R_{V}$ and a lower $u_{n}$ will achieve better performance under the same conditions [20,21].

## 3. Materials and Methods

### 3.1. Principle of Gas Release During Thermal Runaway of Lithium-Ion Battery

The failure of lithium-ion batteries is a complex chemical process, usually involving the decomposition of the electrolyte, the degradation of the electrode materials, and the formation of byproducts. During these processes, certain chemical reactions can produce significant amounts of CO_2_ gas.

SEI decomposition releases CO_2_.

(14) $${\left({CH}_{2}{OCO}_{2}{Li}_{2}\right)}_{2}\to {Li}_{2}{CO}_{3}+C_{2}H_{4}+{CO}_{2}+0.5O_{2}$$

The quantity of CO_2_ generated is directly proportional to the extent of SEI decomposition and can be expressed as:

(15) $$n\left({CO}_{2}\right)=\frac{m_{an}\left(x_{SEI,0}-x_{SEI}\right)}{M\left({\left({CH}_{2}OCO_{2}Li\right)}_{2}\right)}$$

The specific reactions are as follows:

(1) Under overcharging or high-temperature conditions, organic solvents in the electrolyte, such as ethylene carbonate and dimethyl carbonate, can decompose, resulting in the generation of CO_2_;

(16) $$C_{2}H_{5}{OCOOPF}_{4}\to {PF}_{3}O+{CO}_{2}+C_{2}H_{5}F$$

(2) Cathode materials, such as lithium cobalt oxide, can decompose under high-temperature or overcharging conditions. This decomposition can lead to the formation of lithium carbonate, which further breaks down, producing and releasing CO_2_;

(17) $$2{LiCoO}_{2}\to {CoO}_{2}+{Li}_{2}{CO}_{3}$$

(18) $${Li}_{2}{CO}_{3}\to {Li}_{2}O+{CO}_{2}$$

(3) During thermal runaway within a battery, the temperature rapidly escalates, triggering a series of vigorous chemical reactions. Both lithium carbonate and organic lithium salts decompose under these conditions, leading to the release of CO_2_.

(19) $${CH}_{3}{CO}_{2}Li\to {CH}_{3}Li+{CO}_{2}$$

For small lithium battery packs or individual cells, the peak CO_2_ concentration measured during thermal runaway is usually relatively low, typically ranging from several thousand ppm to around 20,000 ppm. In larger battery packs or modules, due to more intense reactions, the peak CO_2_ concentration can be significantly higher, reaching tens of thousands of ppm. In practical applications, the battery management system (BMS) often triggers an alarm when the CO_2_ concentration reaches a certain threshold. For example, when the CO_2_ concentration reaches a few thousand ppm, it can act as an early-warning signal, allowing safety measures to be taken before the battery enters thermal runaway [8,11,12,26,27].

### 3.2. Comparison of Different Pyroelectric Materials

There are two key methods to enhance the accuracy and resolution of NDIR gas sensors: (1) selecting a gas detector with a higher specific detection rate or (2) increasing the power of the infrared source. However, boosting the power of the infrared source may negatively impact the lifespan of the device. As a result, gas detectors with higher detection rates are generally preferred when stronger signals are required. Compared to photodetectors, infrared detectors can operate at room temperature without the need for additional cooling equipment, offer a wider spectral range, and are relatively more cost-effective. These advantages have garnered significant attention for their use in gas detection, as well as in fields like terahertz imaging, medical diagnostics, and spectroscopy.

The pyroelectric coefficient of the PMNT single crystal reaches $p>1.0\times 10^{-3}C/\left(m^{2}K\right)$. The dielectric loss is tan⁡δ<0.1%, and the figure of merit $F_{d}$ reaches up to $40.2\times 10^{-4}C/m^{2}{\;K}^{-1}$, which is far superior to other traditional pyroelectric materials. The following Table 1 lists the performance comparison of commonly used pyroelectric materials [20,21,28,29,30,31].

## 4. Results and Discussion

**Figure 3:**
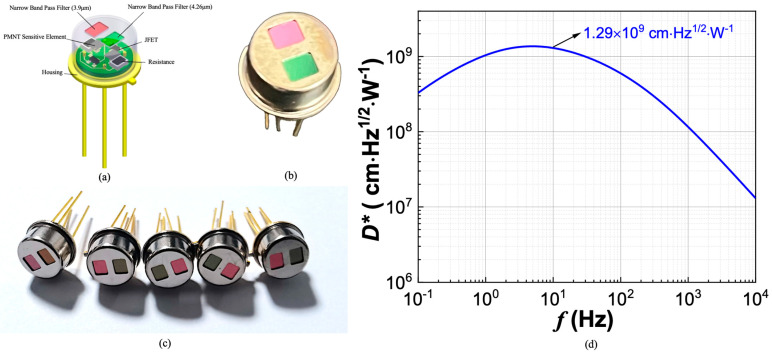
(**a**) Schematic diagram of PMNT CO_2_ gas pyroelectric detector (**b**) Top view of two-channel PMNT CO_2_ gas detector. (**c**) Side view of two-channel PMNT CO_2_ as detector. (**d**) Specific detectivity of PMNT detectors without optical filter.

The pyroelectric CO_2_ gas detector was assembled using a high-performance PMNT single crystal grown at the Shanghai Institute of Ceramics, Chinese Academy of Science [29]. Figure 3a shows the schematic picture of a pyroelectric CO_2_ gas detector. In a pyroelectric detector, the ultra-thin PMNT-sensitive element chip converts infrared radiation into surface charges, which can then be measured as a current. We implemented a “current mode” preamplifier circuit, using an operational amplifier to convert the pyroelectric charges generated on the surface of the sensitive chip into a measurable output voltage signal. In practical applications, pyroelectric IR detectors require high specific detectivity and stable performance under complex and variable external conditions, such as temperature fluctuations and vibration noise, which can be challenging to correct with formulas or algorithms. Using PMNT crystals, we developed both uncompensated and compensated versions of the detector. Compared to the uncompensated detector, the compensated detector has a slightly lower response signal and enhances stability [20]. Although the compensation chip is not directly exposed to IR radiation, it can still absorb a small amount of heat through heat transfer from the nitrogen in the tube cap, thereby reducing the detector’s effective signal. Additionally, the parallel compensation structure increases the equivalent capacitance of the sensitive chip, leading to higher dielectric loss noise and, consequently, an increase in total noise density.

A narrow-band filter with a center wavelength of 4.26 μm and a half-peak width of 180 nm was equipped on the cap as a CO_2_ gas-detective channel, while a narrow-band filter with a center wavelength of 3.9 μm and a half-peak width of 90 nm were equipped as a reference channel. Figure 3b,c show the top and side view of two-channel PMNT-based CO_2_ gas pyroelectric detectors, and the specific detectivity of PMNT detectors without optical filters is shown in Figure 3d. The specific detection rate of PMNT single-crystal pyroelectric infrared detectors is 1.29 × 10^9^ cmHz^1/2^/W@10 Hz, which is 3–4 times higher than commercial LT single-crystal infrared detectors (around 3–4 × 10^8^ cmHz^1/2^/W@10 Hz) [20]. Based on the Lambert–Beer Law in Equation (1) and the detector’s detection limit in Equation (20):

(20) $$I_{0}e^{-q_{1}Lt}-I_{0}e^{-c_{2}Lk}=\frac{1}{D}$$

The detection accuracy formula for NDIR gas detectors is derived as follows:

(21) $$J=\frac{1}{Lk}ln\left(\frac{DI_{0}}{DI_{0}-1}\right)$$

By utilizing this new type of pyroelectric infrared detector, the detection accuracy of NDIR gases can be greatly improved. The data of PMNT and LT pyroelectric detectors with narrow-band optical filters are shown in Table 2. The detectivity in the PMNT pyroelectric detector is about five times that of the LT detector. The accuracy of CO_2_ gases could be calculated by Equation (21), enhanced sharply from 50 ppm level of commercial LT detectors to within 10 ppm of PMNT single-crystal detectors [32]. This advancement enables the rapid and precise detection of minor fluctuations in the CO_2_ concentration, not only improving gas detection accuracy and shortening detection response time but also enabling high-precision online monitoring and analysis of various toxic and harmful gases. The application of this technology in new energy and other fields holds significant economic benefits, which mark a substantial step towards ensuring early detection and prevention of thermal runaway events in electric vehicle batteries.

**Figure 4:**
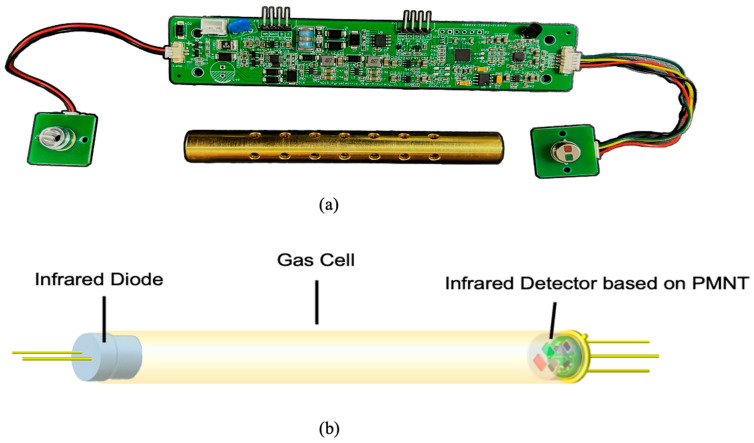
(**a**) Air chamber, IR source, and detector of PMNT NDIR CO_2_ sensor. (**b**) Schematic diagram of NDIR CO_2_ gas sensor detection model.

Figure 4a shows a photo of the CO_2_ NDIR gas sensor used in this experiment, and Figure 4b shows the details of the gas cell model. In the gas sensor, the CO_2_ gas module includes a CO_2_ pyroelectric infrared detector, a self-designed gas chamber, and a MEMS infrared light source. We selected the JSIR-350 MEMS light source (Micro-Hybrid Electronic GmbH, Hermsdorf, Germany) as the infrared source, which offers a broadband infrared spectrum output from 1 to 12 μm, making it suitable for detecting multiple gases with a low power consumption of approximately 100 mW. The gas chamber is 10 cm long, made of brass, and features a gold-plated inner wall.

**Figure 5:**
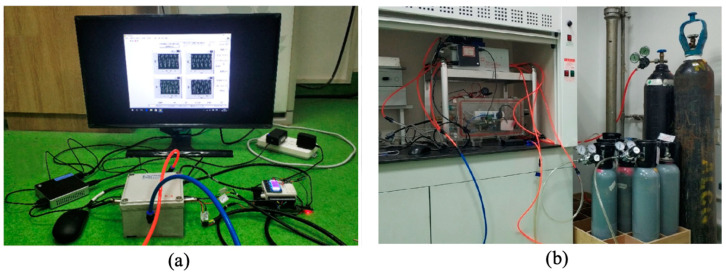
(**a**) Drive and communication circuits of PMNT CO_2_ NDIR gas sensor. (**b**) Photo of testing setup.

Figure 5a shows the drive and communication circuits of a PMNT-based NDIR CO_2_ gas sensor, while Figure 5b is the testing setup. The infrared detector signal is processed through a filtering circuit and an A/D conversion circuit, converting the analog signal into a digital format to output the CO_2_ concentration value. The sensor’s signal voltage was measured using an NI-6002 acquisition board (National Instruments, Austin, TX, USA). With a resolution of 16 bits and an acquisition range of 0–8 V, the board achieves a precision of 122 μV, which is slightly lower than the noise level of the PMNT infrared detector and generally meets the data acquisition requirements for the gas sensor. We used a gas pump and gas chamber to simulate the CO_2_ release process during the thermal runaway of a lithium-ion battery and conducted simulation tests with a self-developed NDIR CO_2_ gas sensor.

To test the sensor, a mixture of CO_2_ and nitrogen (CO_2_-N_2_ mixed gas) was prepared using a Sevenstar CS200 mass flow controller (Beijing Huacheng Electronics Co., Ltd, Beijing, China), with CO_2_ concentrations ranging from 0 ppm to 50,000 ppm. By collecting data from both the detection and reference channels using an NI-6002 acquisition board, an empirical relationship between the CO_2_ concentration and the signal voltage was established based on Lambert–Beer’s law. This empirical formula enables the calculation of the CO_2_ concentration accurately. During the experiment, a CO_2_ concentration–time curve was preset on the computer, and the gas pump controlled the CO_2_ concentration and flow rate over time, thereby simulating the CO_2_ leakage environment associated with lithium-ion battery failure within the gas chamber.

**Figure 6:**
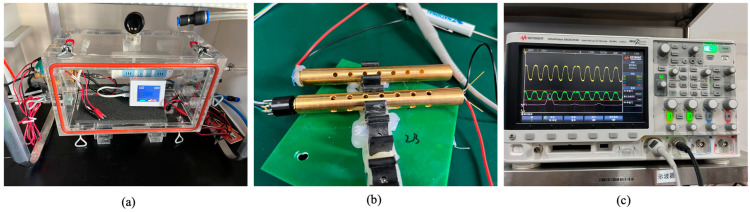
(**a**) Simulation cabin of LIBs CO_2_ release testing. (**b**) Gas chamber. (**c**) Data acquisition system.

In Figure 6, the gas chamber is designed as a cylinder to facilitate the detection and simulation of CO_2_ gas release from LIBs. The cylindrical shape ensures a consistent path for infrared light, reducing scattering and absorption within the chamber, which enhances detection accuracy. The smooth inner walls further improve performance by preventing interference with the infrared light. The chamber’s structure plays a crucial role in determining the gas detection sensitivity. For optimal performance, the infrared light emitted should be as parallel as possible to the chamber’s axis to minimize reflection. Additionally, the inner walls must be smooth enough to prevent infrared light absorption or gas adsorption, ensuring precise detection.

The gas chamber of the NDIR CO_2_ sensor in this design features a circular tube structure with a polished, gold-plated surface to enhance the infrared radiation intensity and prolong its service life. Prior to use, system calibration is necessary, which includes the gas concentration flow control system, power control module, NDIR CO_2_ sensor, and data acquisition, processing, and display system. The CO_2_ concentration range for calibration is 0–50,000 ppm, using a mixture of pure nitrogen and 50,000 ppm CO_2_-N_2_ gas. Calibration of the NDIR module is conducted at room temperature (approximately 25 °C) in 500 ppm intervals. The entire device is sealed with a desiccant to prevent potential interference from water vapor, ensuring accurate gas concentration measurements.

**Figure 7:**
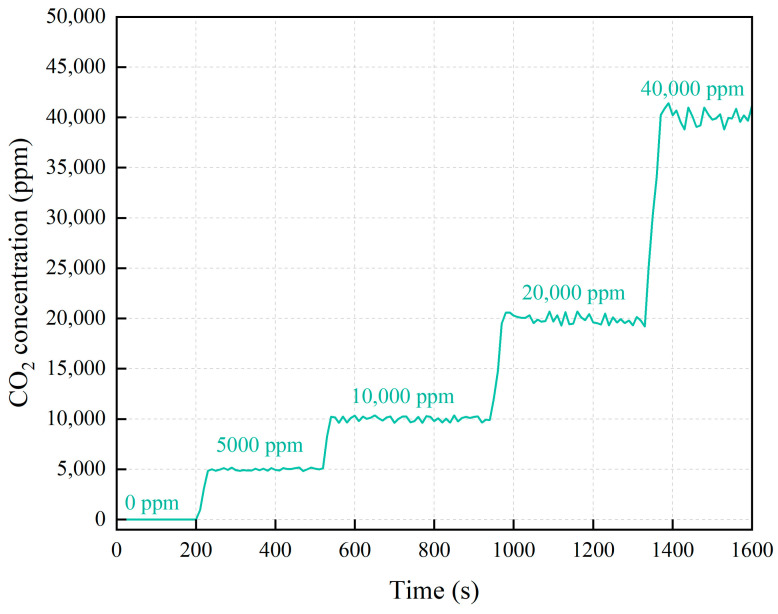
The relationship between CO_2_ concentration and time at different standard concentrations.

**Figure 8:**
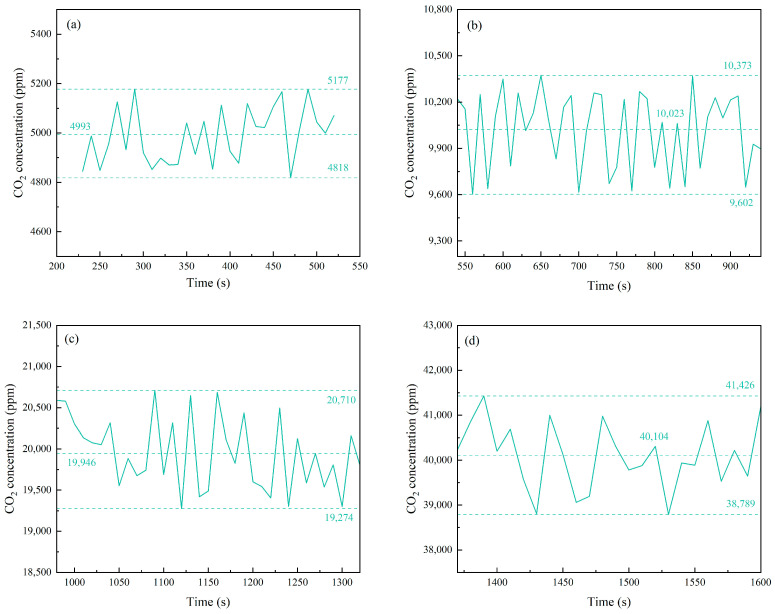
Error values of measured CO_2_ concentrations (**a**) 5000 ppm, (**b**) 10,000 ppm, (**c**) 20,000 ppm, (**d**) 40,000 ppm.

We conducted continuous tests by introducing standard CO_2_ gas at different concentrations and a room temperature of 25 °C. Figure 7 shows the relationship between CO_2_ concentration and time at different standard concentrations, Figure 8 shows the average and error values of measured CO_2_ concentration, and Table 3 summarized the results. For each standard gas concentration, the measured values were 4993 ppm, 10,023 ppm, 19,946 ppm, and 40,104 ppm respectively. The measured concentrations closely matched the standard values, with deviations within ±4.2% of the full-scale (F.S.) calibration range.

**Figure 9:**
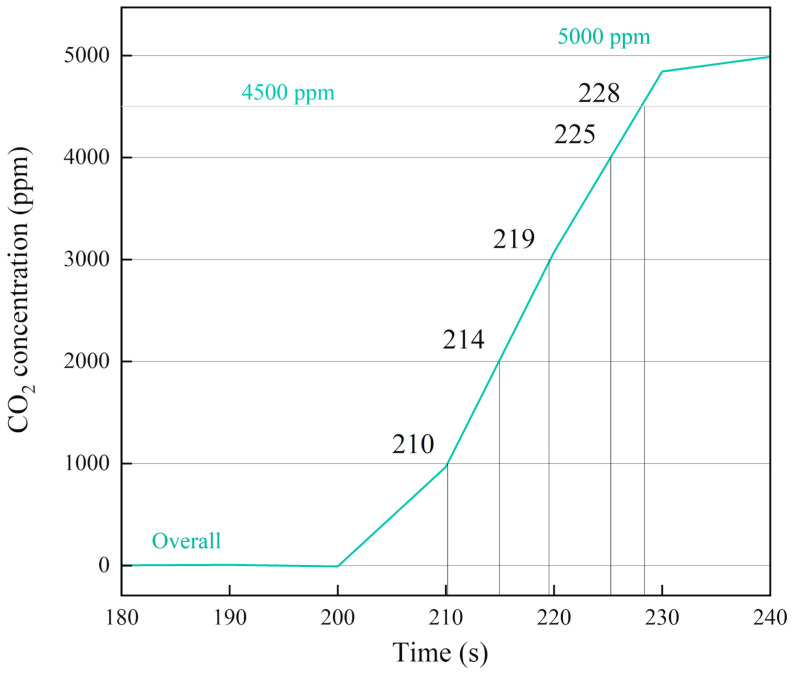
The CO_2_ response time of PMNT NDIR gas sensor at 5000 ppm.

The curve shows the sensor’s reading over time at a standard CO_2_ gas concentration of 5000 ppm. The time it takes for the sensor’s response signal to rise from zero to a certain percentage of the equilibrium concentration is known as the response time. *T*_90_ represents the time required to reach 90% of the target concentration from zero. As shown in Figure 9, after introducing the CO_2_ gas, a noticeable change in concentration is observed within a few seconds. Around 28 s, the sensor reading reaches 90% of the actual concentration, making the *T*_90_ response time 28 s [8,32].

**Figure 10:**
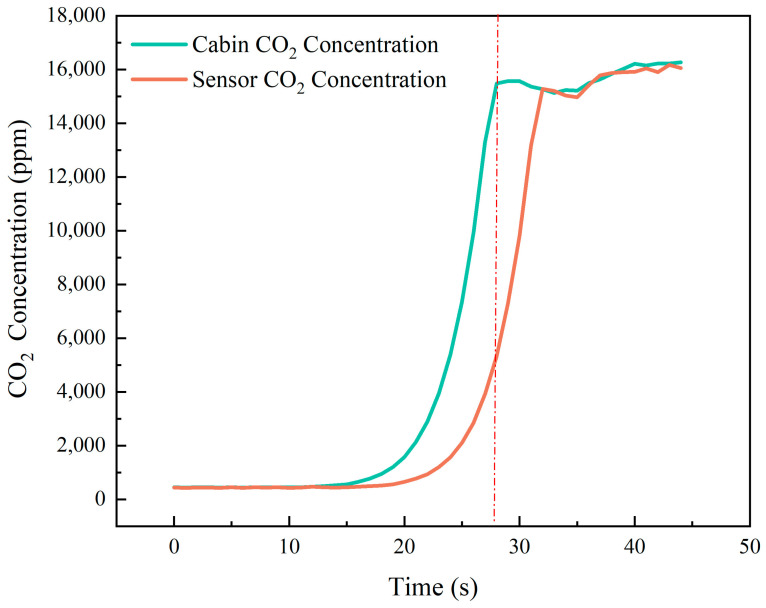
The monitoring effectiveness of PMNT-based NDIR CO_2_ gas sensor.

Using our self-developed NDIR CO_2_ gas sensor, we tested its effectiveness in monitoring the CO_2_ levels during a simulated lithium-ion battery failure. The results, shown in Figure 10, indicate a sensor delay of approximately 5–10 s. When the CO_2_ concentration changes rapidly over a short period (less than 10 s), the sensor responds significantly after about 10 s. As the actual concentration reaches its peak, the sensor reading stabilizes around 5000 ppm, and within 4 s, it closely aligns with the actual concentration. This sensor is, therefore, well-suited for monitoring CO_2_ concentration fluctuations during lithium battery failure. When the concentration reaches approximately 5000 ppm, indicating peak levels, it can serve as an early-warning signal for lithium battery failure.

## 5. Conclusions

We developed a PMNT-based NDIR CO_2_ gas sensor for monitoring lithium battery failure, utilizing a high-performance pyroelectric infrared detector with a PMNT single crystal as the sensitive element. Within a measurement range of 0–50,000 ppm, the sensor’s reading error is less than ±4.2% of the actual value, with a response time (*T*_90_) of 28 s. These specifications surpass the performance of domestic commercial sensors in the same price range. Using this gas sensor, they conducted a simulated lithium battery failure experiment, successfully detecting significant changes in the gas concentration within 10 s, enabling early warning. The test results were favorable.

This study centers on gas detection methodologies, particularly the application of gas sensors in detecting the gases emitted from battery cells within battery packs. Our research also provides a comprehensive analysis of NDIR gas sensors, emphasizing their utility in detecting CO_2_ as the target gas. Through a series of experiments, the study validates the responsiveness and reliability of NDIR CO_2_ sensors.

PMNT single-crystal CO_2_ sensors, known for their high sensitivity, effectively identify thermal runaway in new energy batteries by sensing even the slightest variations in CO_2_ levels. The specific detection rate of PMNT single-crystal pyroelectric infrared detectors is more than four times higher than commercial high-end LT single-crystal infrared detectors. Utilizing this innovative pyroelectric infrared detector enhances the accuracy of gas detection using the NDIR method. It improves the detection accuracy of toxic and harmful gases from the typical 50 ppm level of commercial NDIR modules to within 10 ppm. By implementing narrowband filters to precisely select the infrared light’s detection wavelength, the study enables round-the-clock, real-time online monitoring of toxic and harmful gases, especially CO_2_, in LIBs. This advancement not only enhances gas detection accuracy and reduces response time but also allows for high-precision online monitoring and analysis of various toxic and harmful gases. Consequently, this technology has the potential to find application in the field of new energy and other related industries, yielding substantial economic benefits.

The findings of this research hold substantial significance in addressing critical safety concerns in the electric vehicle industry. Furthermore, it offers valuable references and recommendations to enhance the safety and reliability of electric vehicles.

## Figures and Tables

**Table 1:** Performance comparison of commonly used pyroelectric materials [20,21,28,29,30,31].

Materials	*p*	*ε_r_*	tan *δ*	*F_i_*	*F_v_*	*F_d_*
	10^−4^ Cm^−2^K^−1^	@1 kHz		10^−10^ m/V	m^2^C^−1^	10^−5^ Pa^−1/2^
TGS(35)	5.5	55	2.5%	2.1	0.43	6.1
DTGS(40)	5.5	43	2%	2.3	0.6	8.3
PMNT	15.3	640	0.20%	6.12	0.11	15.3
Mn-PMNT	17.2	660	0.05%	6.88	0.12	40.2
LiTaO_3_	2.3	47	0.05%	0.72	0.17	15.7
PZT	3.3	714	0.2%		1.22	0.02
BST	35	5000	1.0%		13.72	0.03

**Table 2:** Properties of PMNT single-crystal and LT pyroelectric detectors with optical filters.

Types of Detectors Types of Optical Filters		PMNT		LiTaO_3_	
		CO_2_	Refer	CO_2_	Refer
*u_n_*	μV·Hz^−1/2^	82.0	82.1	31.8	35.1
*R_V_*	V/W	9331	14,419	729.0	616.0
*NEP*	μW	0.009	0.006	0.044	0.057
D*	cm·Hz^1/2^·W^−1^	2.28 × 10^7^	3.51 × 10^7^	4.58 × 10^6^	3.51 × 10^6^

**Table 3:** CO_2_ measured concentrations at different standard concentrations.

Standard CO_2_ Concentration (ppm)	Measured Concentration (ppm)	Measured Error (ppm)	F.S. (%)
5000	4993	+184/−175	±3.7
10,000	10,023	+350/−421	±4.2
20,000	19,946	+764/−672	±3.8
40,000	40,104	+1322/−1315	±3.3

## Data Availability

Data are contained within the article.
